# Supplementary material for: Explainable multi-task learning for multi-modality biological data analysis
Source: Nat Commun. 2023 May 3;14:2546. doi: 10.1038/s41467-023-37477-x (PMC10156823; doi:10.1038/s41467-023-37477-x)
Supplement: Supplementary file 1 — Supplementary information [file 41467_2023_37477_MOESM1_ESM.pdf]

Supplementary Information for

Explainable multi-task learning for multi-modality biological data analysis

Xin Tang[1,2,6], Jiawei Zhang[3,6], Yichun He[1,2,6], Xinhe Zhang[1], Zuwan Lin[4], Sebastian Partarrieu[1], Emma Bou Hanna[1], Zhaolin Ren[1], Hao Shen[1], Yuhong Yang[3], Xiao Wang[2,5], Na Li[1], Jie Ding[3]* & Jia Liu[1]*

[1]John A. Paulson School of Engineering and Applied Sciences, Harvard University, Boston, MA 02134, USA

[2]Broad Institute of MIT and Harvard, Cambridge, MA 02142, USA.

[3]School of Statistics, University of Minnesota Twin Cities, Minneapolis, MN 55455, USA.

[4]Department of Chemistry and Chemical Biology, Harvard University, Cambridge, MA 02138, USA

[5]Department of Chemistry, MIT, Cambridge, MA 02139, USA.

[6]These authors contributed equally: Xin Tang, Jiawei Zhang, Yichun He.

*e-mail: dingj@umn.edu, jia_liu@seas.harvard.edu

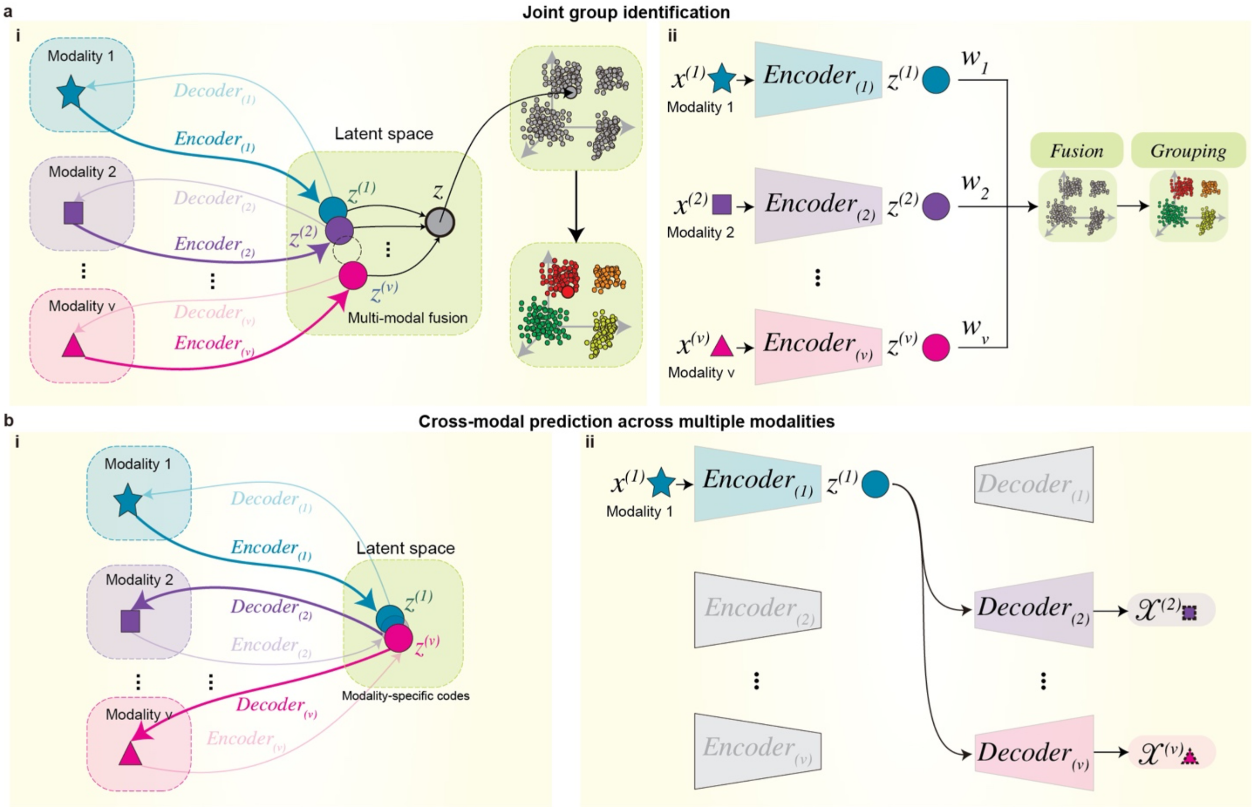

Supplementary Fig. 1: Tasks in UnitedNet and the multi-task learning procedure. a, An example of a joint group identification task. (**i**) Modality 1, modality 2, and other modalities are encoded to the latent space as modality-specific codes through corresponding encoders. The modality-specific latent codes are then fused as a shared latent code. The shared latent code is then used for unsupervised/supervised group identification through the grouping module. (**ii**) The network structure used for the joint group identification task in (**i**). **b**, An example of a cross-modal prediction task. (**i**) Modality 1 is encoded to the latent space as modality-specific codes by encoder 1. The modality-specific codes are then decoded to the space of modality 2 and modality v through decoder 2 and decoder v. (**ii**) The network structure used for the cross-modal prediction task in (**i**).

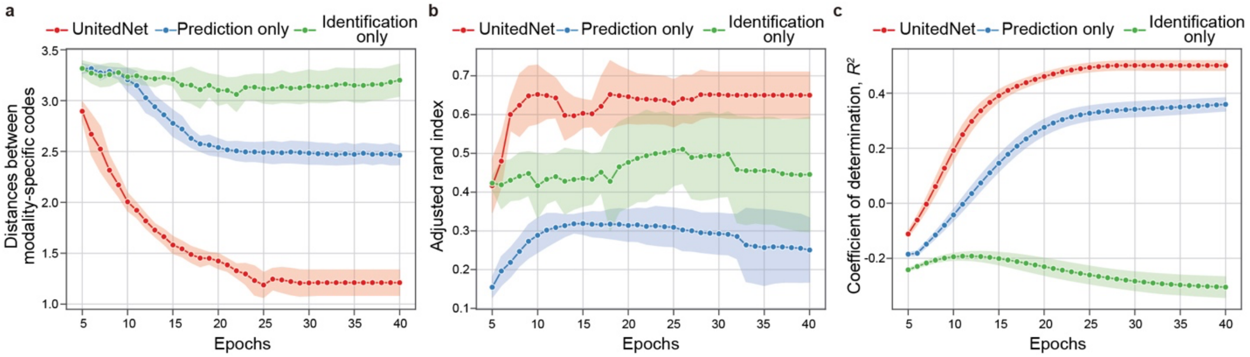

Supplementary Fig. 2: Ablation analysis of UnitedNet on a simulated Dyngen dataset. a, Line plots showing the change of the average distance of modality-specific codes from the latent space of UnitedNet with different numbers of training epochs. **b**, Line plots showing the change in the performance of joint group identification that is measured by the adjusted rand index with different numbers of training epochs. **c**, Line plots showing the change in the performance of cross-modal prediction as measured by the coefficient of determination, R^2, with different numbers of training epochs. Error bands show mean ± SD over 5-fold cross-validation for panels **a**-**c**. Source data are provided as a Source Data file.

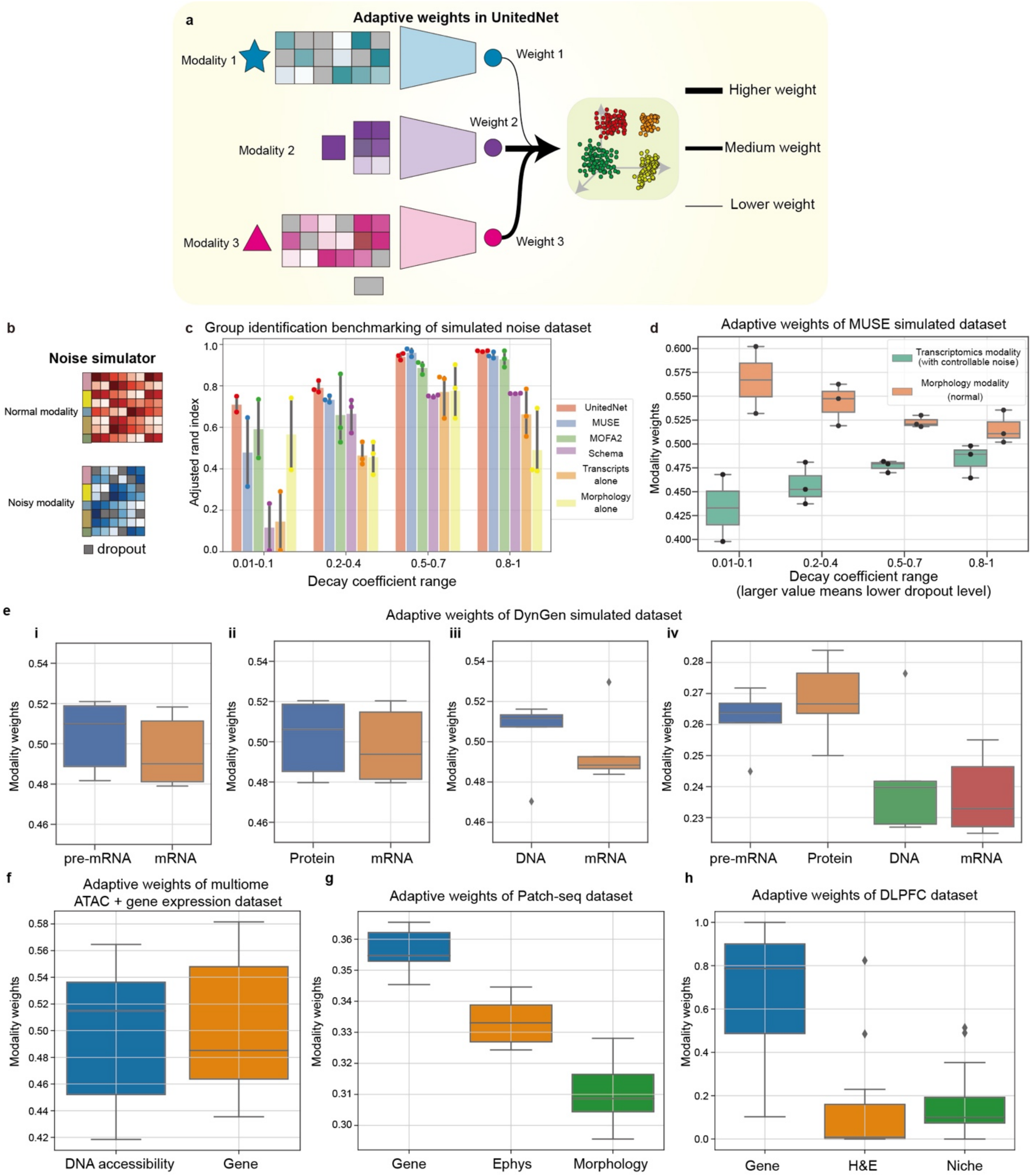

Supplementary Fig. 3: UnitedNet validation with simulated dataset and adaptive weighting scheme. a, Schematics showing the adaptive weighting scheme of the joint group identification task in UnitedNet. Each modality was encoded to the latent space as modality-specific codes through corresponding encoders. Then, the modality-specific latent codes were adaptively weighted based on the noise level and fused as a shared latent code. The shared latent code is then used for unsupervised/supervised group identification through the grouping module. **b**, Schematics of the MUSE simulator simulating the dropout effect in the transcriptomics data[27]. The transcriptomics modality is simulated with a controllable dropout level. **c**, Barplots comparing the

accuracy of cell typing between UnitedNet and other methods as the dropout level changes. Data are represented as mean ± SD. **d**, Boxplot showing the modality weight learned in UnitedNet for different modalities of the simulated data in (**b**). n = 2 dropout levels ranging from 0.01-0.1, n = 3 dropout levels ranging from 0.2-0.4, n = 3 dropout levels ranging from 0.5-0.7, n = 3 dropout levels ranging from 0.8-1. **e-h**, Boxplots showing the modality weight learned in UnitedNet for different modalities of the simulated DynGen dataset (**e**), multiome ATAC+gene expression BMMCs dataset (**f**), Patch-seq GABAergic neuron dataset (**g**), and spatial DLPFC dataset (**h**). n = 5 folds cross-validation for panel **e**. n = 13 folds cross-validation for panel **f**. n = 10 folds cross-validation for panel **g**. n = 12 folds cross-validation for panel **h**. Box, 75% and 25% quantiles. Line, median. Whisker, the maxima/minima or to the median ± 1.5× IQR. Source data are provided as a Source Data file.

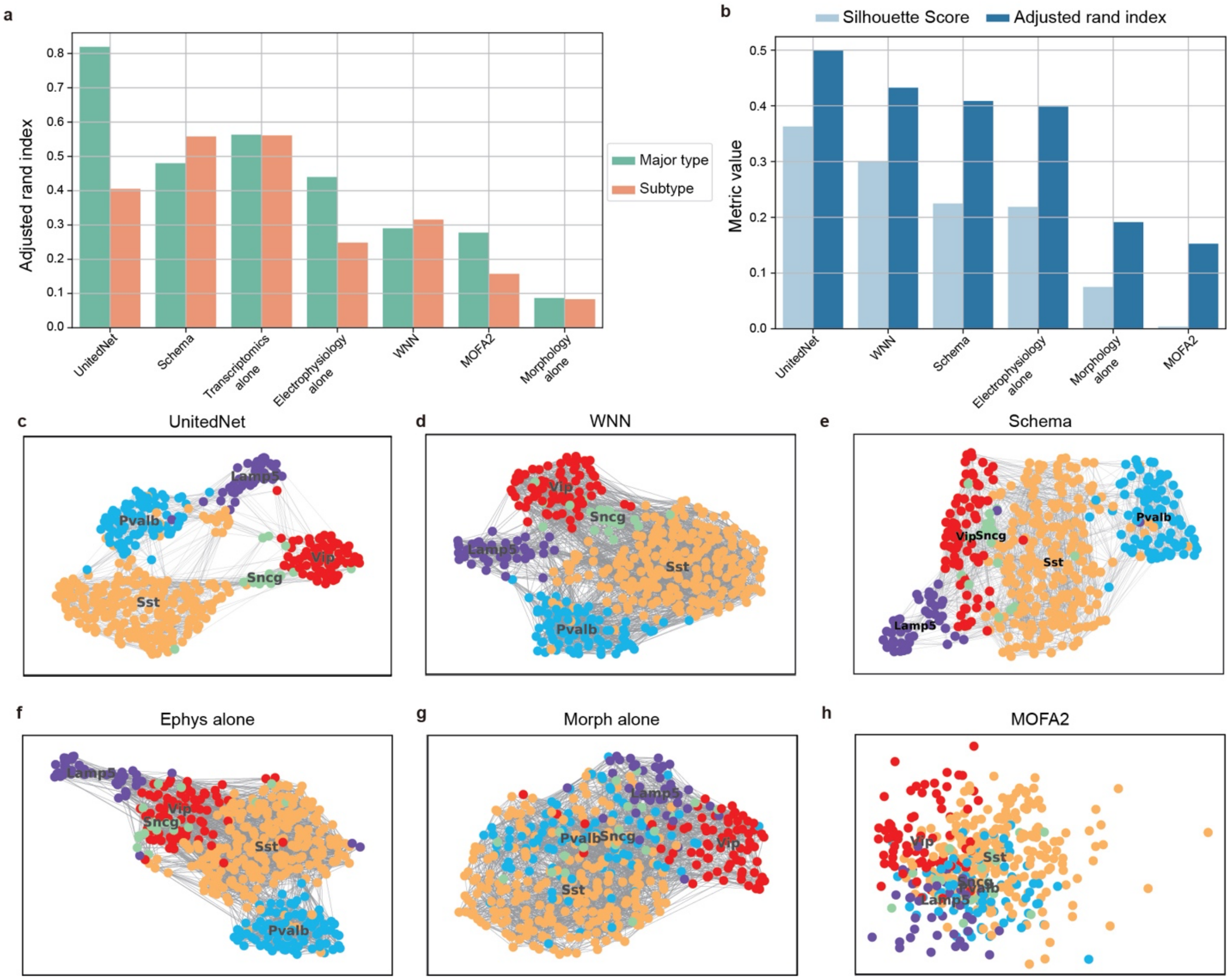

Supplementary Fig. 4: Fusion methods benchmarking on the Patch-seq dataset. **a**, Barplot showing performance comparison between UnitedNet and state-of-the-art methods including single-modality Leiden clustering, Schema, WNN, and MOFA on the Patch-seq GABAergic neuron dataset using transcriptomics, electrophysiology, and morphology modalities for MET cell type identification. The benchmarking was conducted on both major- and sub-MET types identification tasks. **b**, Barplot showing performance comparison between UnitedNet and state-of-the-art methods including single-modality Leiden clustering, Schema, WNN, and MOFA on the Patch-seq GABAergic neuron dataset using electrophysiology and morphology modalities for cell type identification. The separability of cell clusters defined by electrophysiology and morphology modalities in the latent space were evaluated against the transcriptomics-defined cell types and quantified using the silhouette score. **c-h**, The latent space visualization by UMAP of UnitedNet (**c**), WNN (**d**), Schema (**e**), electrophysiology data alone (**f**), and morphology alone (**g**), and MOFA2 (**h**). The latent codes are colored to represent different cell type labels. Source data are provided as a Source Data file.

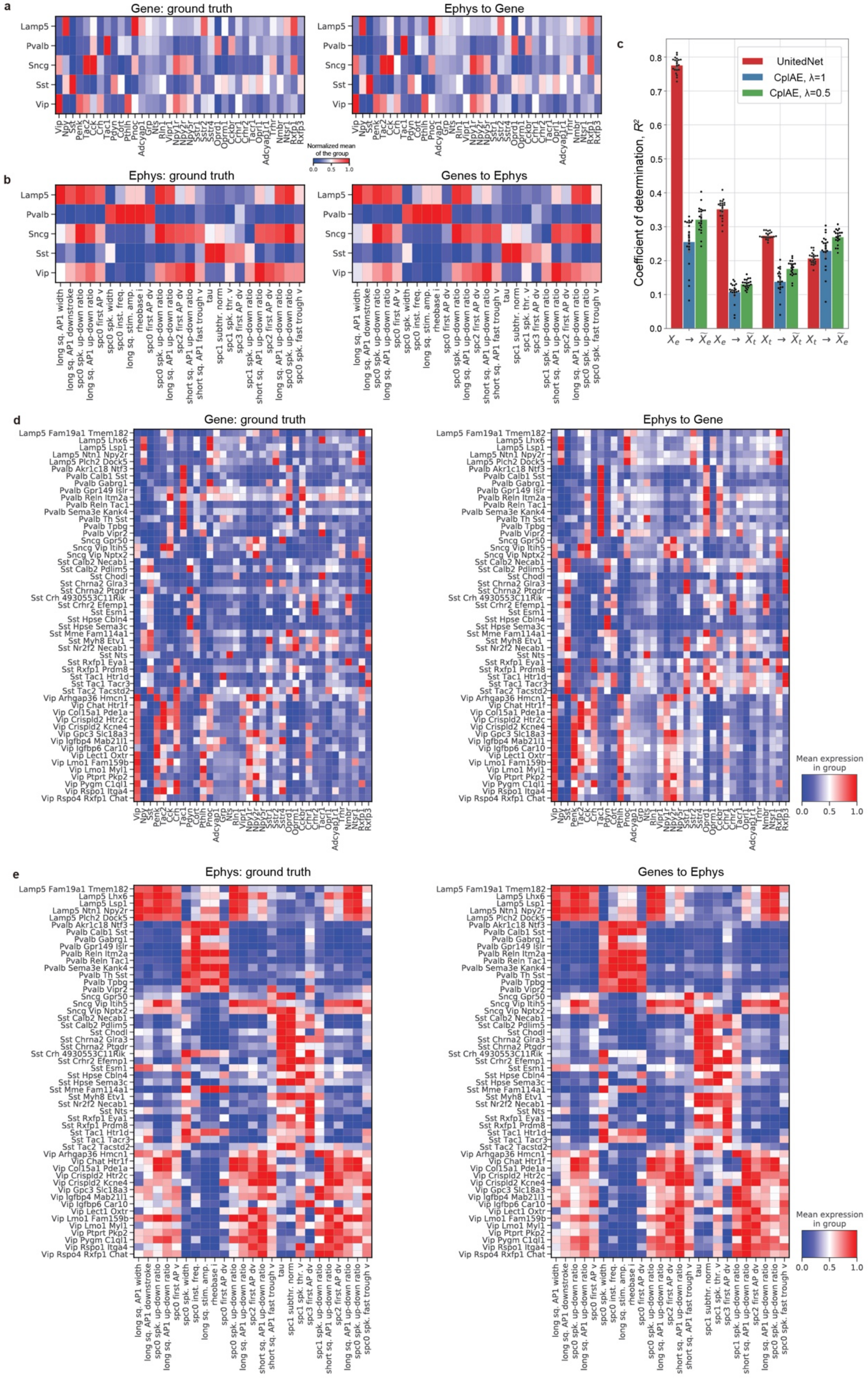

a
Gene: ground truth
Ephys to Gene
Lamp5
Pvalb
Sncg
Sst
Vip
Vip Npy Sst Penk Tac2 Cck Crh Tac1 Pdyn Cort Pthlh Pnoc Adcyap1 Grp Nts Rln1 Vipr1 Npy1r Npy2r Npy5r Sstr1 Sstr2 Sstr4 Oprd1 Oprm1 Cckbr Crhr1 Crhr2 Tacr1 Oprl1 Adcyap1r1 Trhr Nmbr Ntsr1 Rxfp1 Rxfp3
Normalized mean of the group
0.0 0.5 1.0
b
Ephys: ground truth
Genes to Ephys
long sq. AP1 width
long sq. AP1 downstroke
spc0 spk. up-down ratio
long sq. AP1 up-down ratio
spc0 first AP v
spc0 spk. width
spc0 inst. freq.
long sq. stim. amp.
rheobase i
spc0 first AP dv
spc0 spk. up-down ratio
long sq. AP1 up-down ratio
spc2 first AP dv
short sq. AP1 up-down ratio
short sq. AP1 fast trough v
tau
spc1 subthr. norm
spc1 spk. thr. v
spc3 first AP dv
spc1 spk. up-down ratio
short sq. AP1 up-down ratio
spc2 first AP dv
long sq. AP1 up-down ratio
spc0 spk. up-down ratio
spc0 spk. fast trough v
c
UnitedNet
CplAE, λ=1
CplAE, λ=0.5
Coefficient of determination, R^2
0.0 0.1 0.2 0.3 0.4 0.5 0.6 0.7 0.8
$X_e \rightarrow \overline{X}_e$ $X_e \rightarrow \overline{X}_t$ $X_t \rightarrow \overline{X}_t$ $X_t \rightarrow \overline{X}_e$
d
Gene: ground truth
Ephys to Gene
Lamp5 Fam19a1 Tmem182
Lamp5 Lhx6
Lamp5 Lsp1
Lamp5 Ntn1 Npy2r
Lamp5 Plch2 Dock5
Pvalb Akr1c18 Ntf3
Pvalb Calb1 Sst
Pvalb Gabrg1
Pvalb Gpr149 Islr
Pvalb Reln Itm2a
Pvalb Reln Tac1
Pvalb Sema3e Kank4
Pvalb Th Sst
Pvalb Tpbg
Pvalb Vipr2
Sncg Gpr50
Sncg Vip Itih5
Sncg Vip Nptx2
Sst Calb2 Necab1
Sst Calb2 Pdlim5
Sst Chodl
Sst Chrna2 Glra3
Sst Chrna2 Ptgdr
Sst Crh 4930553C11Rik
Sst Crhr2 Efemp1
Sst Esm1
Sst Hpse Cbln4
Sst Hpse Sema3c
Sst Mme Fam114a1
Sst Myh8 Etv1
Sst Nr2f2 Necab1
Sst Nts
Sst Rxfp1 Eya1
Sst Rxfp1 Prdm8
Sst Tac1 Htr1d
Sst Tac1 Tacr3
Sst Tac2 Tacstd2
Vip Arhgap36 Hmcn1
Vip Chat Htr1f
Vip Col15a1 Pde1a
Vip Crispld2 Htr2c
Vip Crispld2 Kcne4
Vip Gpc3 Slc18a3
Vip Igfbp4 Mab21l1
Vip Igfbp6 Car10
Vip Lect1 Oxtr
Vip Lmo1 Fam159b
Vip Lmo1 Myl1
Vip Ptprt Pkp2
Vip Pygm C1ql1
Vip Rspo1 Itga4
Vip Rspo4 Rxfp1 Chat
Mean expression in group
0.0 0.5 1.0
e
Ephys: ground truth
Genes to Ephys
Mean expression in group
0.0 0.5 1.0

Supplementary Fig. 5: Cross-modal prediction result on electrophysiology and transcriptomics modality from the Patch-seq dataset. a-b, Heatmap comparing cross-modal predicted gene expression and electrophysiological features averaged over annotated major cell types with the ground truth. **c,** Bar and dot plots comparing within- and cross-modal prediction performances between two modalities (transcriptomics and electrophysiology) by CplAE with different values of hyperparameter λ and UnitedNet. n = 20 folds cross validation. Error bars show mean ± SD over 20 folds cross-validation. **d-e,** Heatmap comparing cross-modal predicted gene expression and electrophysiological features averaged over annotated detailed transcriptomics cell types with the ground truth. Source data are provided as a Source Data file.

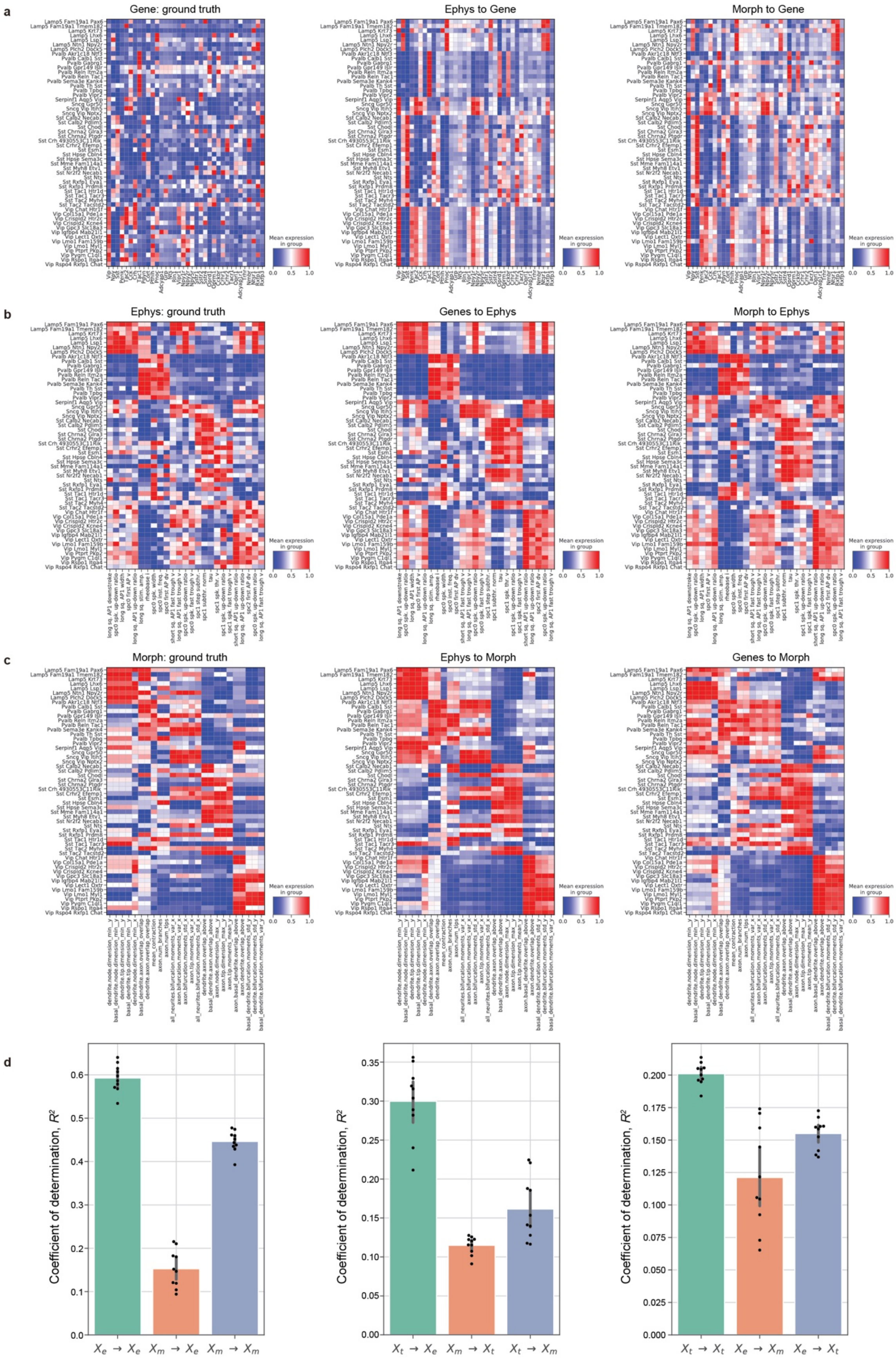
a
Gene: ground truth
Ephys to Gene
Morph to Gene
b
Ephys: ground truth
Genes to Ephys
Morph to Ephys
c
Morph: ground truth
Ephys to Morph
Genes to Morph
Mean expression in group
0.0 0.5 1.0
d
Coefficient of determination, R^2
$X_e \to X_e$
$X_m \to X_e$
$X_m \to X_m$
$X_t \to X_e$
$X_m \to X_t$
$X_t \to X_m$
$X_t \to X_t$
$X_e \to X_m$
$X_e \to X_t$

Supplementary Fig. 6: Prediction results of Patch-seq with detailed transcriptomics cell type. a-c, Heatmap comparing cross-modal predicted gene expression, electrophysiological and morphological features averaged over annotated detailed transcriptomics cell types with the ground truth. **d**, Bar and dot plots of within- and cross-modal prediction performances among three modalities (transcriptomics, morphology, and electrophysiology) by UnitedNet. n = 10 folds cross validation. Error bars show mean ± SD over 10 folds cross-validation. Source data are provided as a Source Data file.

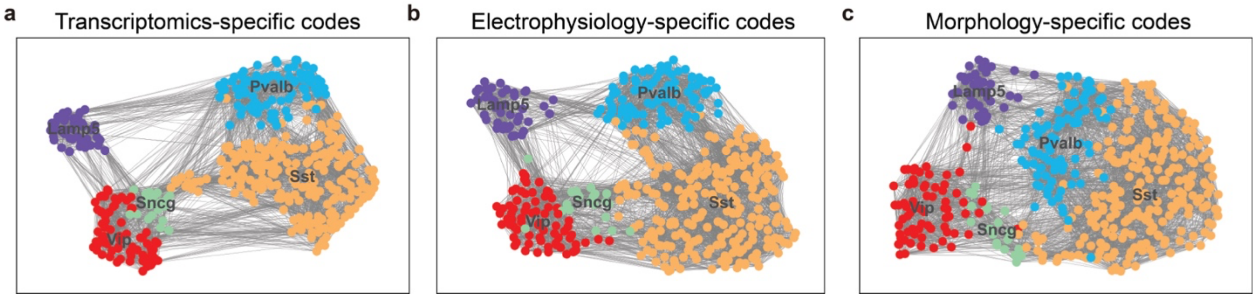

Supplementary Fig. 7: Three-modality analyses of the Patch-seq GABAergic neuron enabled by UnitedNet. a-c, Latent space visualization of the modality-specific codes of the transcriptomics (**a**), electrophysiology (**b**), and morphology (**c**) in the Patch-seq GABAergic neuron dataset. All codes are color-coded by the joint morphology-electrophysiology-transcriptomics (MET)-types defined by the previous study. Source data are provided as a Source Data file.

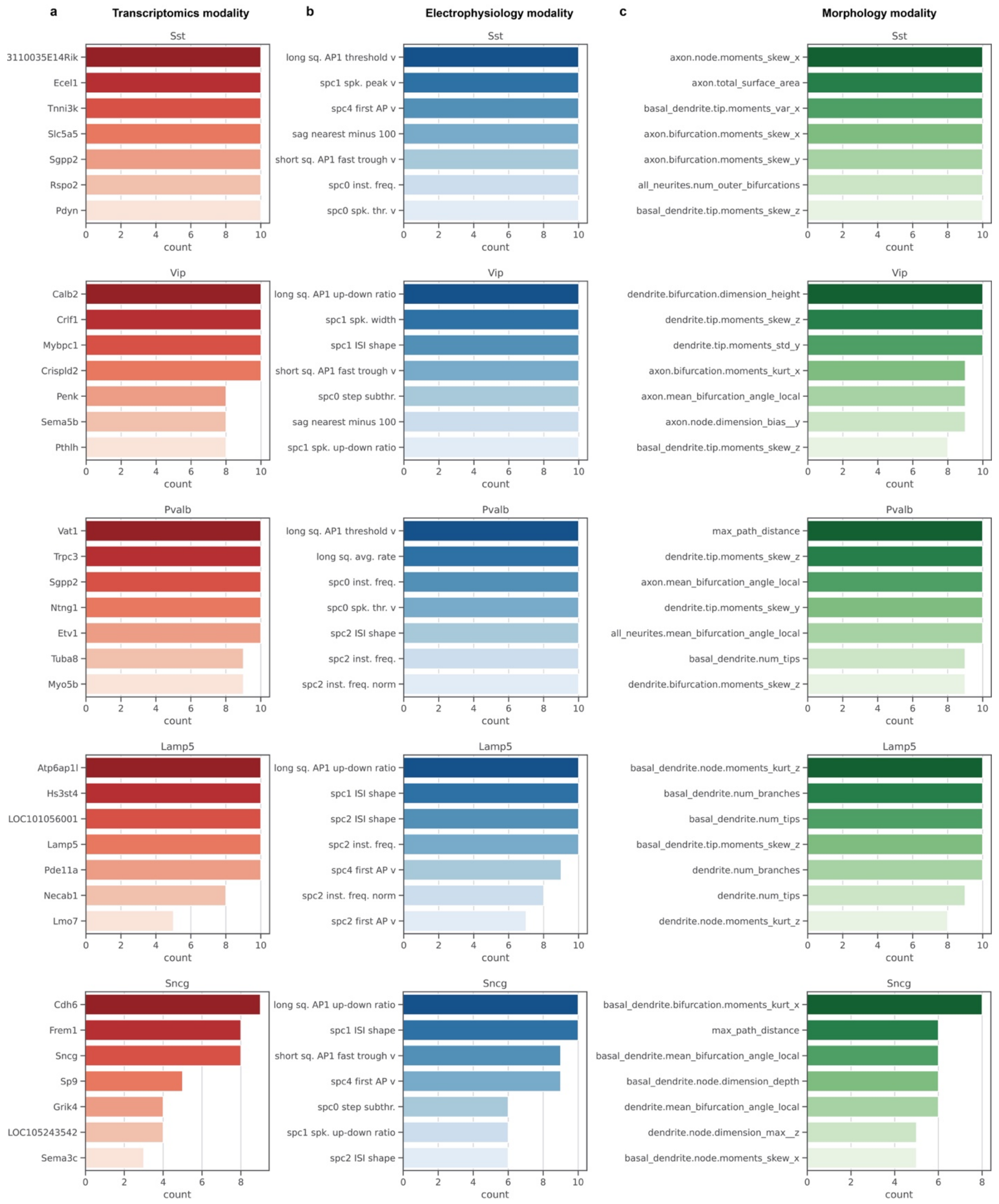

Supplementary Fig. 8: Robustness validation of explainable learning by dissecting a fixed UnitedNet model trained with Patch-seq GABAergic neuron dataset. a-c, Barplot showing the frequency of SHAP-identified neuron-type specific genes (**a**), electrophysiological features (**b**), and morphological features (**c**). First, the UnitedNet was trained on the fixed Patch-seq

GABAergic neuron dataset. Then, the Patch-seq GABAergic neuron dataset was evenly divided into 10 folds and each fold was used as input of SHAP to calculate the Shapley values. For each fold, the modality features with the top 7 largest Shapley values were selected. Finally, we counted the frequency of the selected features across the 10 folds and chose the top 7 most frequent features where the results are presented in the barplots. Source data are provided as a Source Data file.

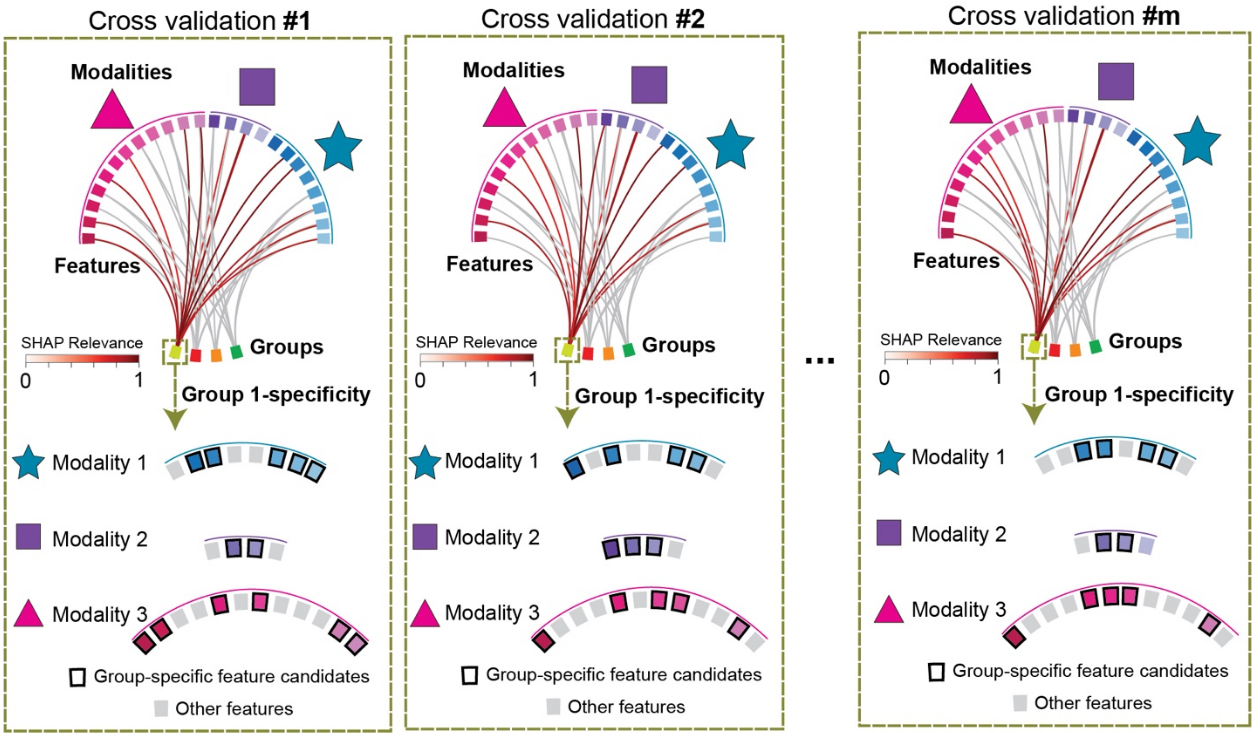

Supplementary Fig. 9: Robustness validation of UnitedNet with explainable learning. Schematics showing the process of robustness test of explainable learning in UnitedNet using cross-validations. The UnitedNet models trained with different model initialization and random samples for the calculation of SHAP were independently dissected by the explainable learning method. Each dissection can generate a set of cell-type-specific features. The robustness of the identified features was defined as their repetition rate in all cross-validations.

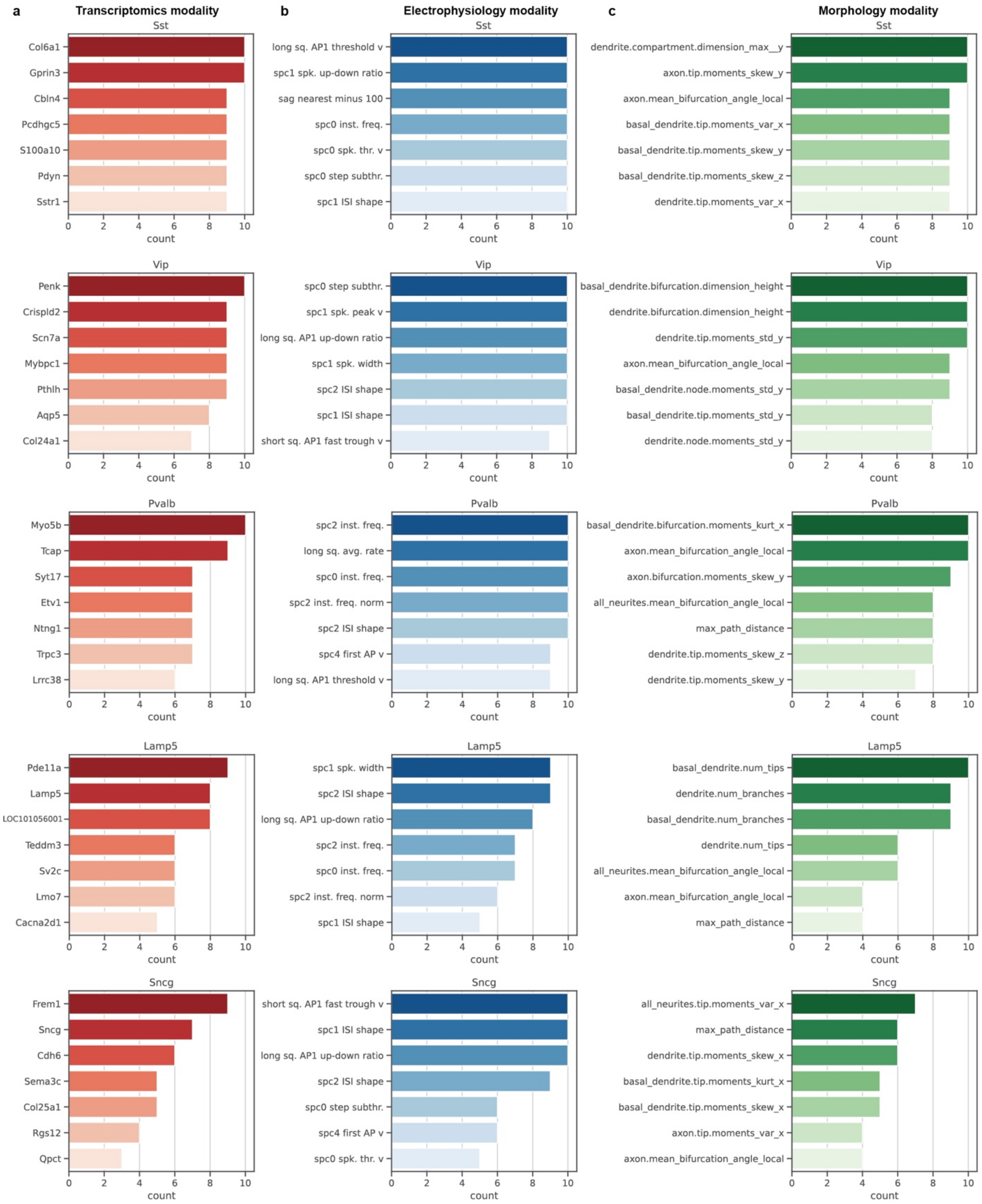

Supplementary Fig. 10: Robustness validation of explainable learning by dissecting the UnitedNet models across multiple folds of cross-validation in Patch-seq GABAergic neuron dataset. a-c, Barplot showing the frequency of SHAP-identified neuron-type specific genes (**a**), electrophysiological features (**b**), and morphological features (**c**) in 10 cross-validation replications. The top 7 most frequent features were shown in each frequency barplot. The

procedure is similar with the one for **Supplementary Fig. 8** except that we in turn set aside a validation fold from the 10 folds cross-validation, retrain UnitedNet on the remaining nine folds, and evaluate SHAP values on the validation fold. Source data are provided as a Source Data file.

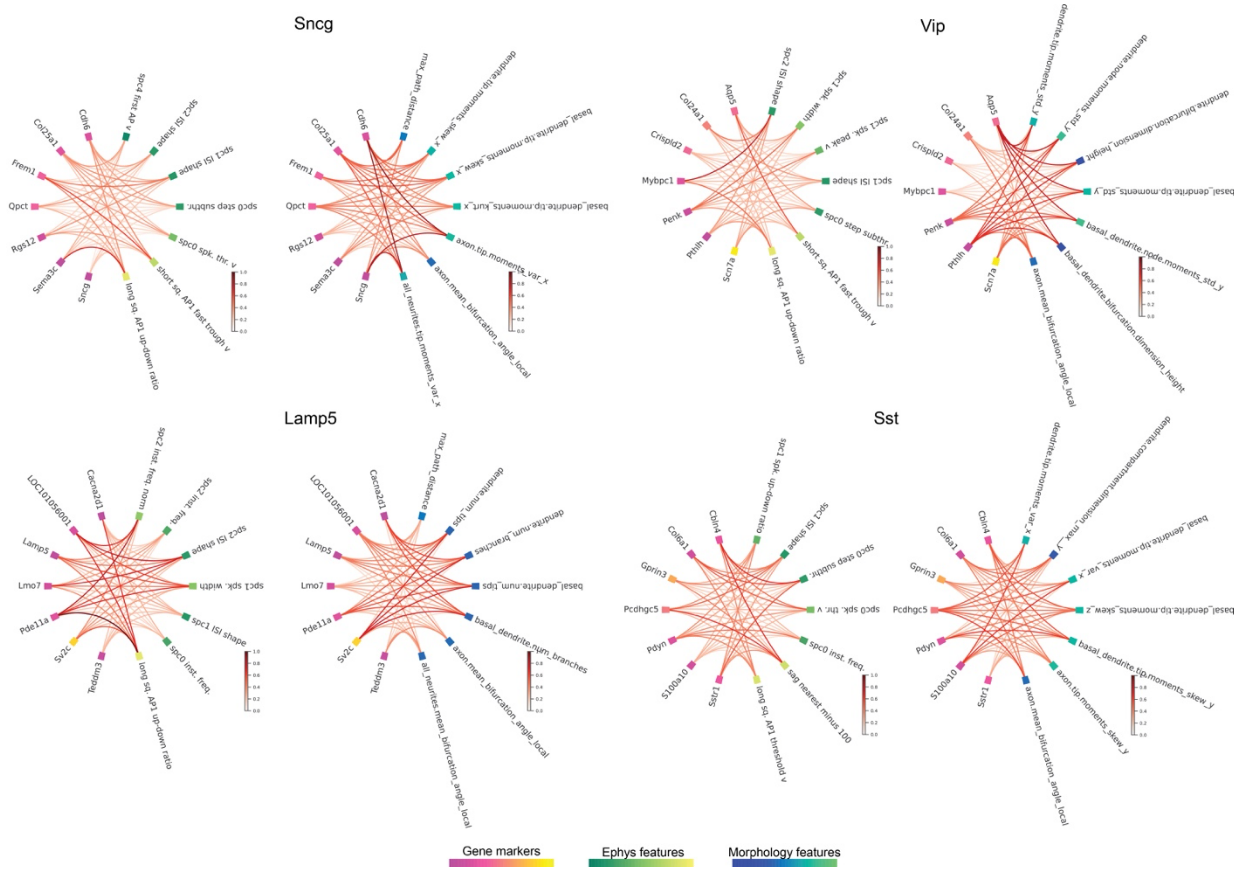

Supplementary Fig. 11: Feature-to-feature relevance with cell-type specificity in Patch-seq data. Chord diagram showing the feature-to-feature relevance for each GABAergic neuron type calculated by dissecting the trained UnitedNet with explainable machine learning. The relevance values are min-max normalized to 0-1. Source data are provided as a Source Data file.

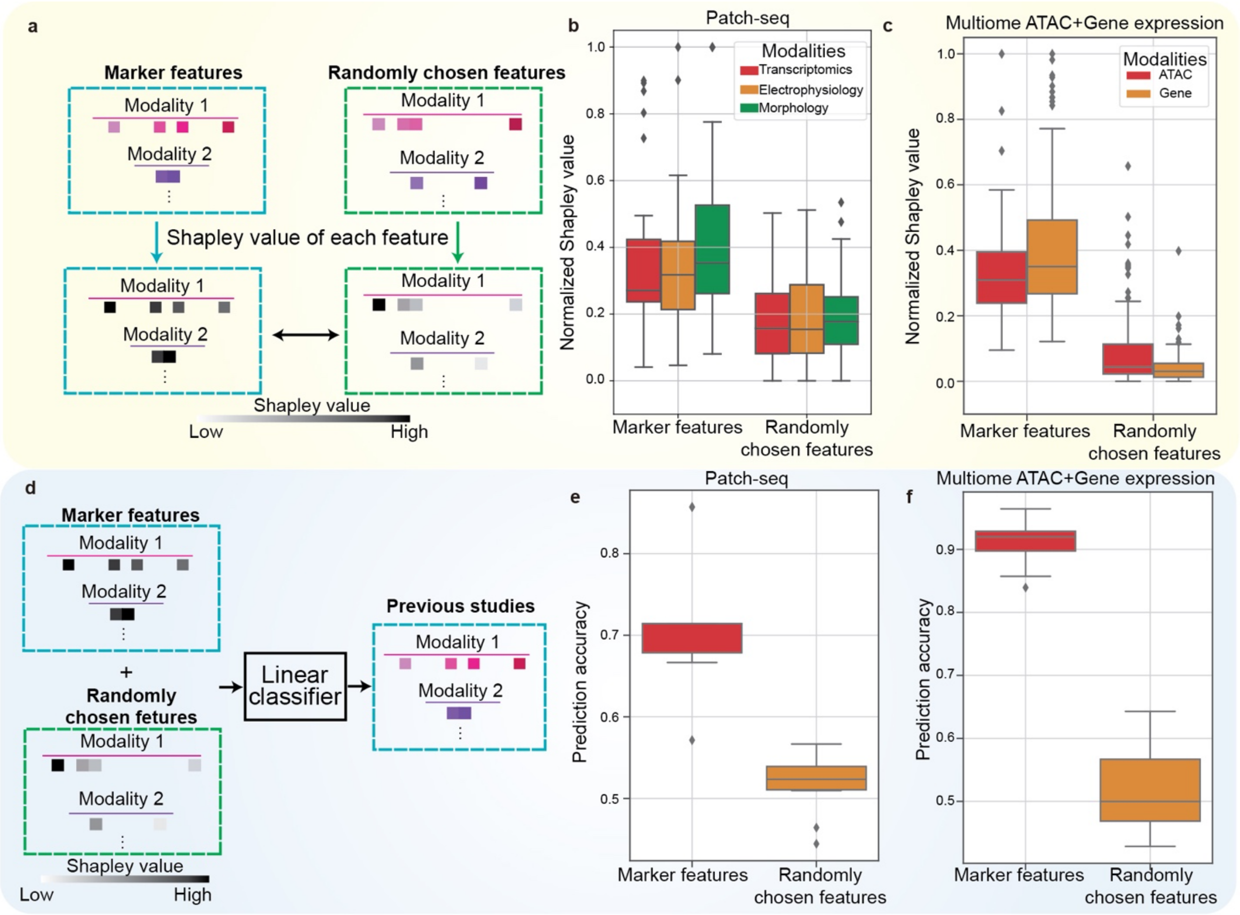

Supplementary Fig. 12: Effectiveness validation of explainable learning in UnitedNet. a, Schematics showing the comparison of Shapley values for previous studies and randomly selected features. The Shapley value was calculated for each feature by dissecting the trained model. **b-c**, Barplot showing the Shapley value of marker features from the previous studies and randomly selected features in the Patch-seq GABAergic neuron dataset (**b**) and multiome ATAC+gene expression BMMCs dataset (**c**). The Shapley values of the same modality are normalized to the range of [0,1] for comparison. n = 35 modality features over 5 cell types for panel **b**. n = 140 modality features over 7 cell types for panel **c**. **d**, Schematics of using a linear classifier to predict whether a feature is a marker feature based on its Shapley value. Randomly selected features were used as a control in the linear classifier. **e-f**, Barplot showing the Shapley value of marker features from the previous studies and randomly selected features in the Patch-seq GABAergic neuron dataset (**e**) and multiome ATAC+gene expression BMMCs dataset (**f**). The Shapley values of the same modality are min-max normalized to the range of [0,1] for comparison. n = 10 folds cross validation for panel **e**. n = 13 folds cross validation for panel **f**. Box, 75% and 25% quantiles. Line, median. Whisker, the maxima/minima or to the median ± 1.5× IQR. Source data are provided as a Source Data file.

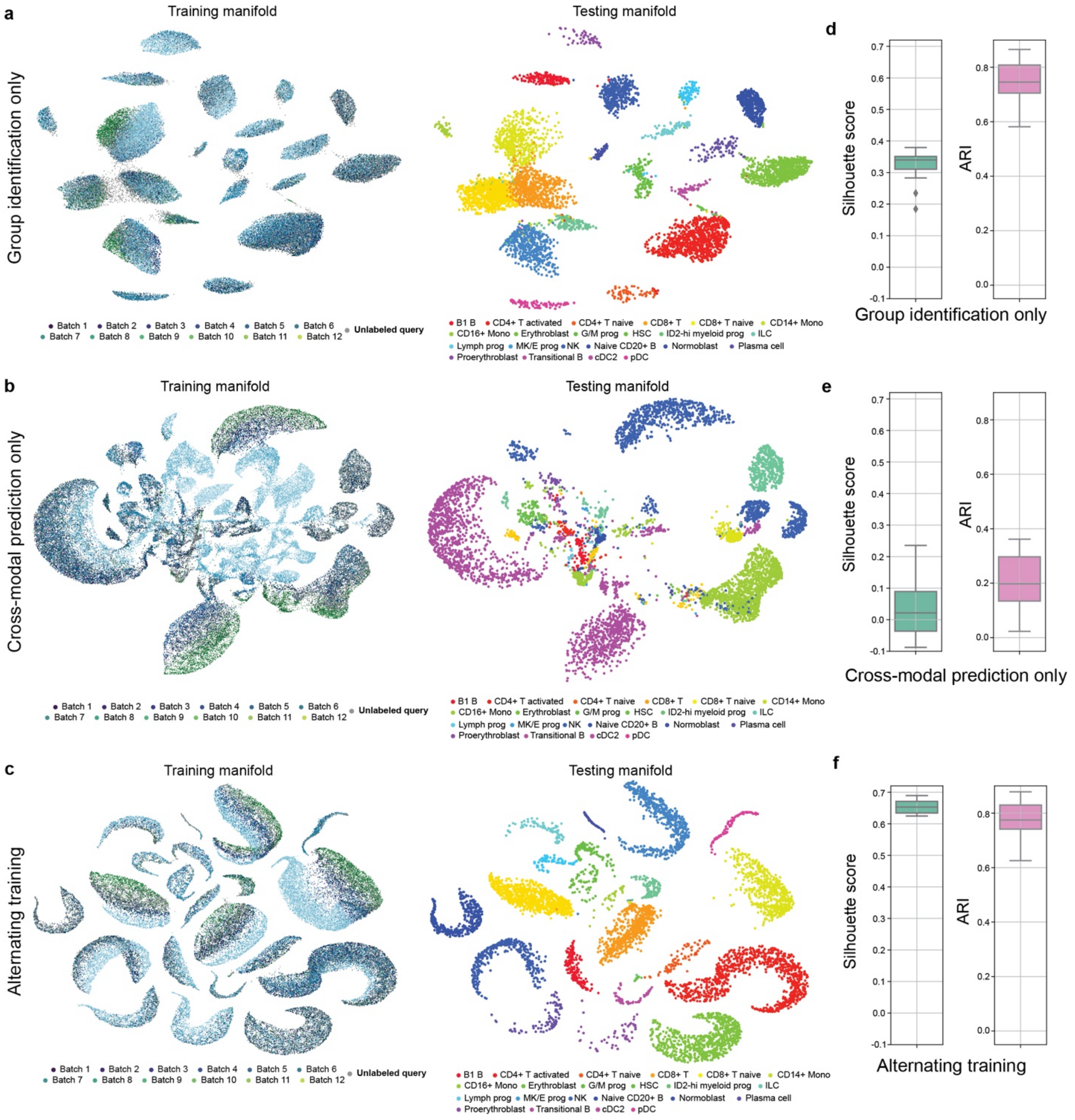

Supplementary Fig. 13: Batch effect correction in multiome ATAC+gene expression BMMCs dataset with UnitedNet. a-c, The UMAP latent space visualizations showing shared latent codes of UnitedNet trained with group identification task only (**a**), cross-modal prediction task only (**b**), and alternating training (**c**) of the 13-batch multiome ATAC+gene expression BMMCs dataset. In the training manifold, the latent codes are colored by different batches. In the testing manifold, the latent codes are colored by cell-type annotations. **d-e**, Boxplot showing the comparison of testing manifold separability in terms of Silhouette score and testing data group identification accuracy in terms of ARI between the UnitedNet trained by joint group identification task only (**d**), cross-modal prediction task only (**e**), and alternating training (**f**) for the multiome

ATAC+gene expression dataset. n = 13 folds cross validation for panel **d**-**f**. Box, 75% and 25% quantiles. Line, median. Whisker, the maxima/minima or to the median ± 1.5× IQR. Source data are provided as a Source Data file.

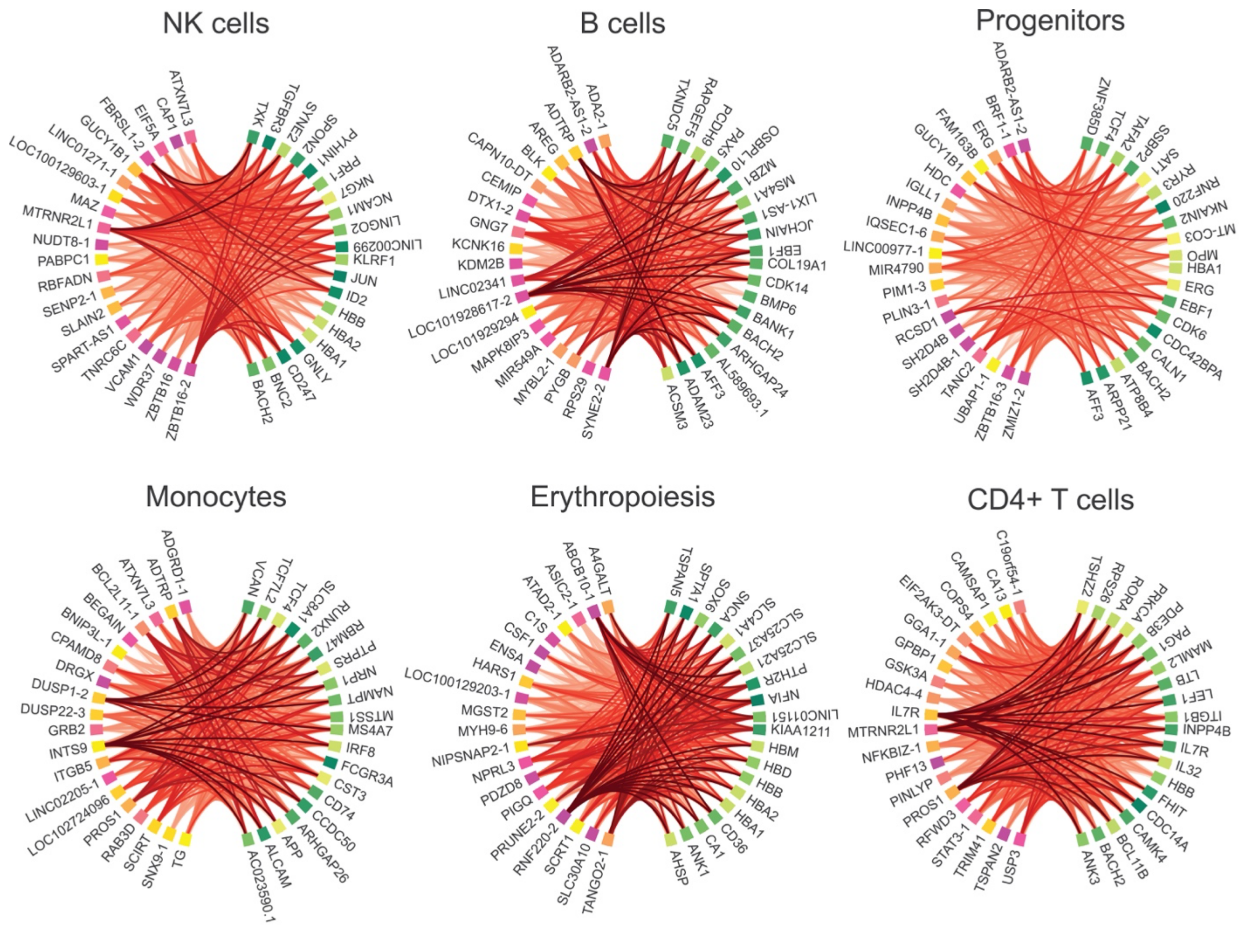

Supplementary Fig. 14: Feature-to-feature relevance with cell-type specificity in multiome ATAC+gene expression data. Chord diagram showing the feature-to-feature relevance for each BMMC cell type calculated by dissecting the trained UnitedNet with explainable machine learning. The relevance values are min-max normalized to 0-1. Source data are provided as a Source Data file.

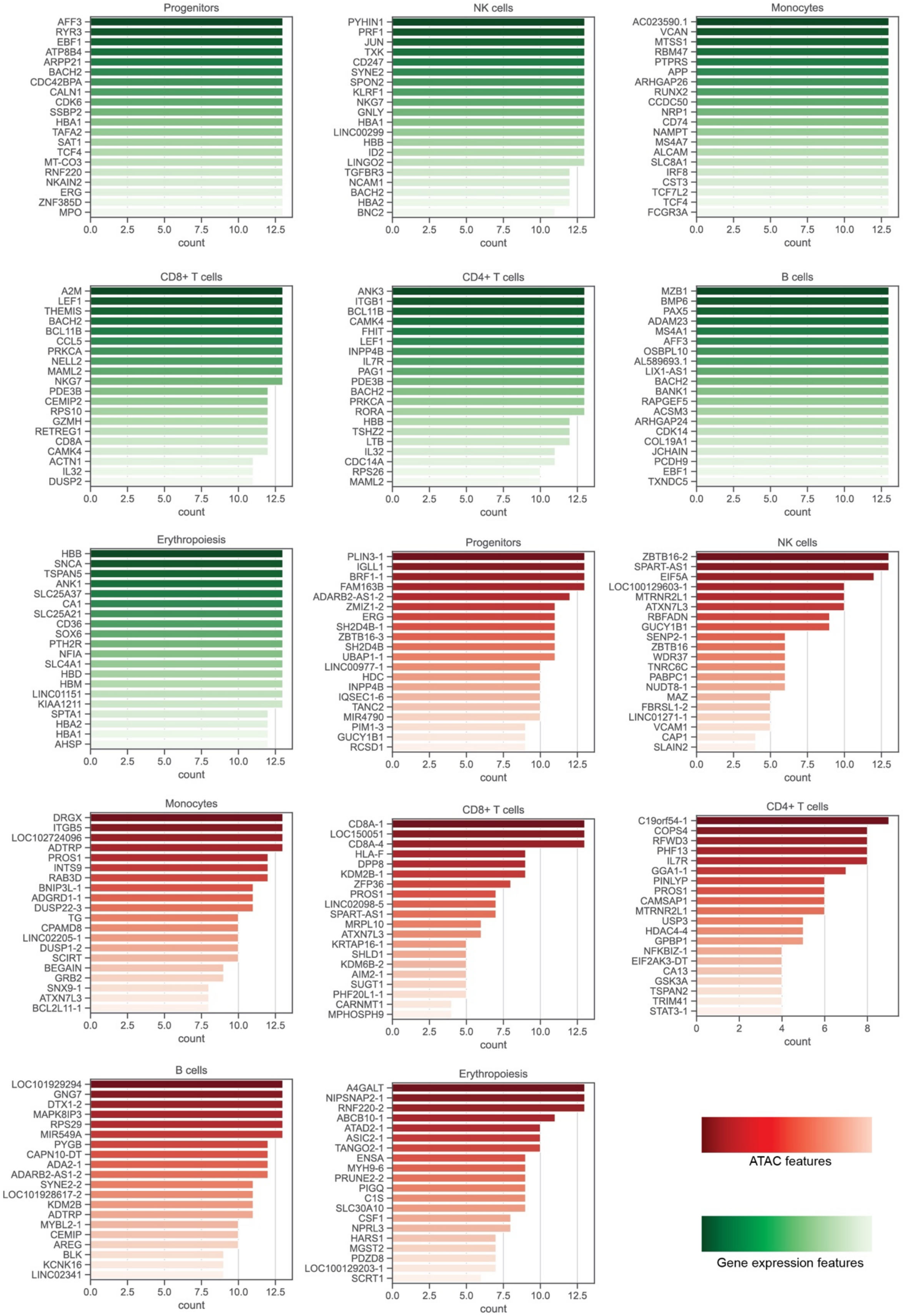
Progenitors
AFF3
RYR3
EBF1
ATP8B4
ARPP21
BACH2
CDC42BPA
CALN1
CDK6
SSBP2
HBA1
TAFA2
SAT1
TCF4
MT-CO3
RNF220
NKAIN2
ERG
ZNF385D
MPO
count
NK cells
PYHIN1
PRF1
JUN
TXK
CD247
SYNE2
SPON2
KLRF1
NKG7
GNLY
HBA1
LINC00299
HBB
ID2
LINGO2
TGFBR3
NCAM1
BACH2
HBA2
BNC2
count
Monocytes
AC023590.1
VCAN
MTSS1
RBM47
PTPRS
APP
ARHGAP26
RUNX2
CCDC50
NRP1
CD74
NAMPT
MS4A7
ALCAM
SLC8A1
IRF8
CST3
TCF7L2
TCF4
FCGR3A
count
CD8+ T cells
A2M
LEF1
THEMIS
BACH2
BCL11B
CCL5
PRKCA
NELL2
MAML2
NKG7
PDE3B
CEMIP2
RPS10
GZMH
RETREG1
CD8A
CAMK4
ACTN1
IL32
DUSP2
count
CD4+ T cells
ANK3
ITGB1
BCL11B
CAMK4
FHIT
LEF1
INPP4B
IL7R
PAG1
PDE3B
BACH2
PRKCA
RORA
HBB
TSHZ2
LTB
IL32
CDC14A
RPS26
MAML2
count
B cells
MZB1
BMP6
PAX5
ADAM23
MS4A1
AFF3
OSBPL10
AL589693.1
LIX1-AS1
BACH2
BANK1
RAPGEF5
ACSM3
ARHGAP24
CDK14
COL19A1
JCHAIN
PCDH9
EBF1
TXNDC5
count
Erythropoiesis
HBB
SNCA
TSPAN5
ANK1
SLC25A37
CA1
SLC25A21
CD36
SOX6
PTH2R
NFIA
SLC4A1
HBD
HBM
LINC01151
KIAA1211
SPTA1
HBA2
HBA1
AHSP
count
Progenitors
PLIN3-1
IGLL1
BRF1-1
FAM163B
ADARB2-AS1-2
ZMIZ1-2
ERG
SH2D4B-1
ZBTB16-3
SH2D4B
UBAP1-1
LINC00977-1
HDC
INPP4B
IQSEC1-6
TANC2
MIR4790
PIM1-3
GUCY1B1
RCSD1
count
NK cells
ZBTB16-2
SPART-AS1
EIF5A
LOC100129603-1
MTRNR2L1
ATXN7L3
RBFADN
GUCY1B1
SENP2-1
ZBTB16
WDR37
TNRC6C
PABPC1
NUDT8-1
MAZ
FBRSL1-2
LINC01271-1
VCAM1
CAP1
SLAIN2
count
Monocytes
DRGX
ITGB5
LOC102724096
ADTRP
PROS1
INTS9
RAB3D
BNIP3L-1
ADGRD1-1
DUSP22-3
TG
CPAMD8
LINC02205-1
DUSP1-2
SCIRT
BEGAIN
GRB2
SNX9-1
ATXN7L3
BCL2L11-1
count
CD8+ T cells
CD8A-1
LOC150051
CD8A-4
HLA-F
DPP8
KDM2B-1
ZFP36
PROS1
LINC02098-5
SPART-AS1
MRPL10
ATXN7L3
KRTAP16-1
SHLD1
KDM6B-2
AIM2-1
SUGT1
PHF20L1-1
CARNMT1
MPHOSPH9
count
CD4+ T cells
C19orf54-1
COPS4
RFWD3
PHF13
IL7R
GGA1-1
PINLYP
PROS1
CAMSAP1
MTRNR2L1
USP3
HDAC4-4
GPBP1
NFKBIZ-1
EIF2AK3-DT
CA13
GSK3A
TSPAN2
TRIM41
STAT3-1
count
B cells
LOC101929294
GNG7
DTX1-2
MAPK8IP3
RPS29
MIR549A
PYGB
CAPN10-DT
ADA2-1
ADARB2-AS1-2
SYNE2-2
LOC101928617-2
KDM2B
ADTRP
MYBL2-1
CEMIP
AREG
BLK
KCNK16
LINC02341
count
Erythropoiesis
A4GALT
NIPSNAP2-1
RNF220-2
ABCB10-1
ATAD2-1
ASIC2-1
TANGO2-1
ENSA
MYH9-6
PRUNE2-2
PIGQ
C1S
SLC30A10
CSF1
NPRL3
HARS1
MGST2
PDZD8
LOC100129203-1
SCRT1
count
ATAC features
Gene expression features

Supplementary Fig. 15: Robustness validation of UnitedNet with explainable learning in multiome ATAC+gene expression BMMCs dataset. Barplot showing the frequency of SHAP-identified cell-type-specific genes (reds) and DNA accessibilities (greens) in 13 cross-validation replications. The top 20 most frequent features were shown in each frequency barplot. The procedure is similar with the one for Supplementary Fig. 10 except that we in turn set aside a validation fold from the 13 folds cross-validation, retrain UnitedNet on the remaining 12 folds, and evaluate SHAP values on the validation fold in the multiome ATAC+gene expression BMMCs dataset. Source data are provided as a Source Data file.

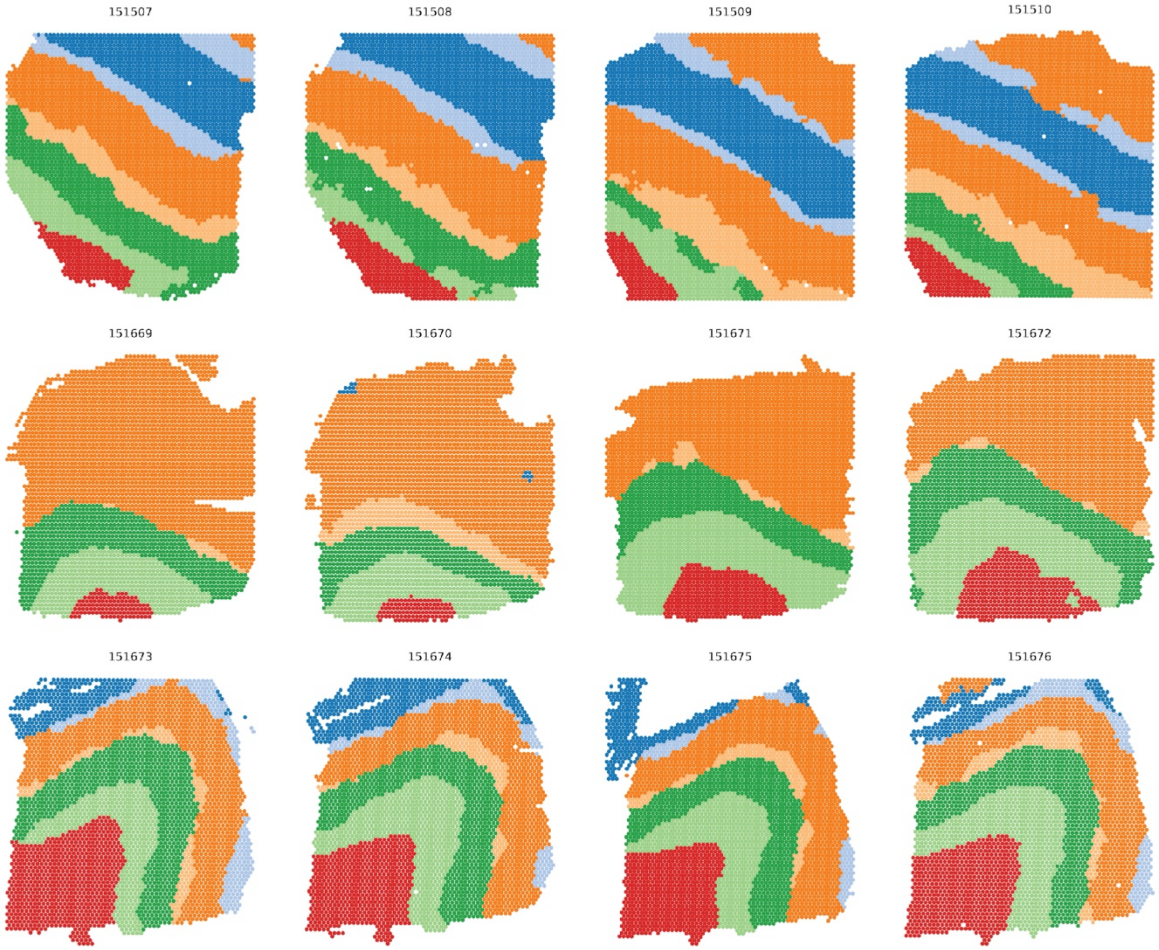

Supplementary Fig. 16: Annotation transferred result for DLPFC using UnitedNet. Brain slices colored with transferred annotation. The spots of each measured brain slice are colored by tissue region labels predicted by UnitedNet. For each sample, UnitedNet is trained on the remaining 11 batches and is tested on this batch.

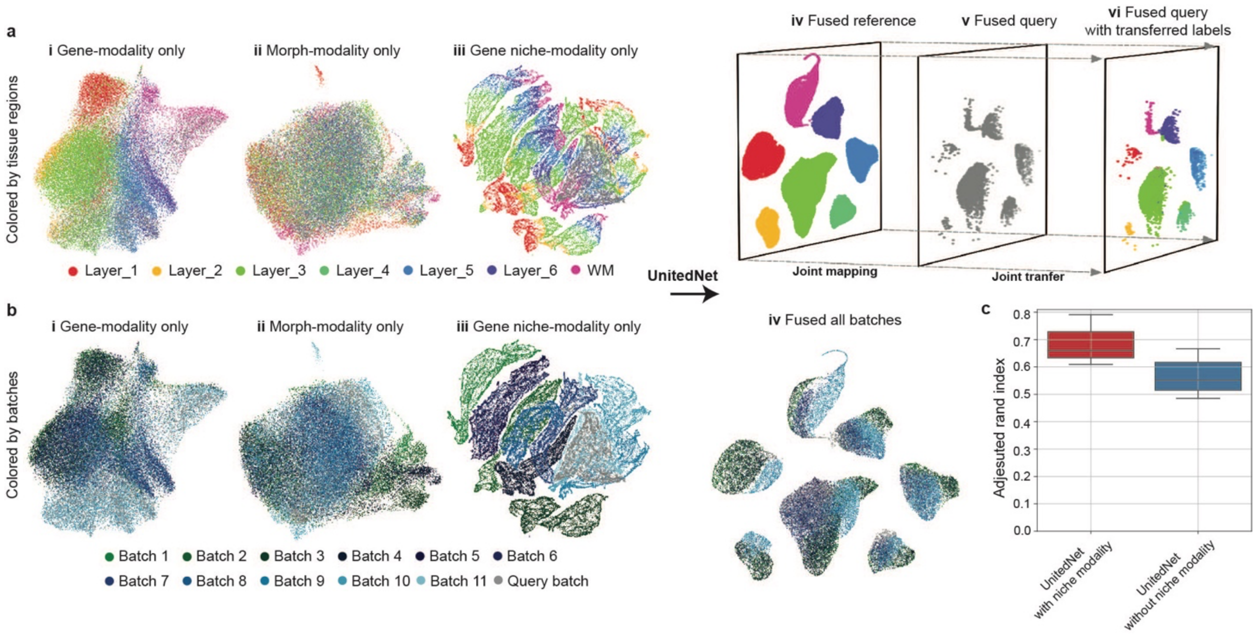

Supplementary Fig. 17: UnitedNet enables supervised group identification in the spatial-omics dataset. a, Supervised joint group identification enabled by UnitedNet. The UMAP latent space visualizations show gene expression (i), morphology (ii), gene expression niche (iii), and shared latent codes of UnitedNet (iv-vi) of the 12-batch DLPFC dataset. UnitedNet fuses the shared latent codes from three modalities and groups the codes based on tissue-region annotations (iv). Then, it projects the unlabeled query batch to the learned shared latent space (v), transferring the label of the reference to the shared latent codes of the unlabeled query batch to complete the annotation transfer (vi). The latent codes are colored by tissue-region annotations. **b**, The UMAP latent space visualizations show gene expression (i), morphology (ii), gene expression niche (iii), and shared latent codes of UnitedNet of the 12-batch DLPFC dataset (iv). The latent codes are colored by different batches. **c**, Boxplot showing the ablation analysis of cell niche modality. UnitedNet trained with gene expression, morphology, and cell niche modality shows better performance in joint group identification task than the UnitedNet trained with gene expression and morphology modality. n = 12 folds cross-validation. Box, 75% and 25% quantiles. Line, median. Whisker, the maxima/minima or to the median ± 1.5× IQR. Source data are provided as a Source Data file.

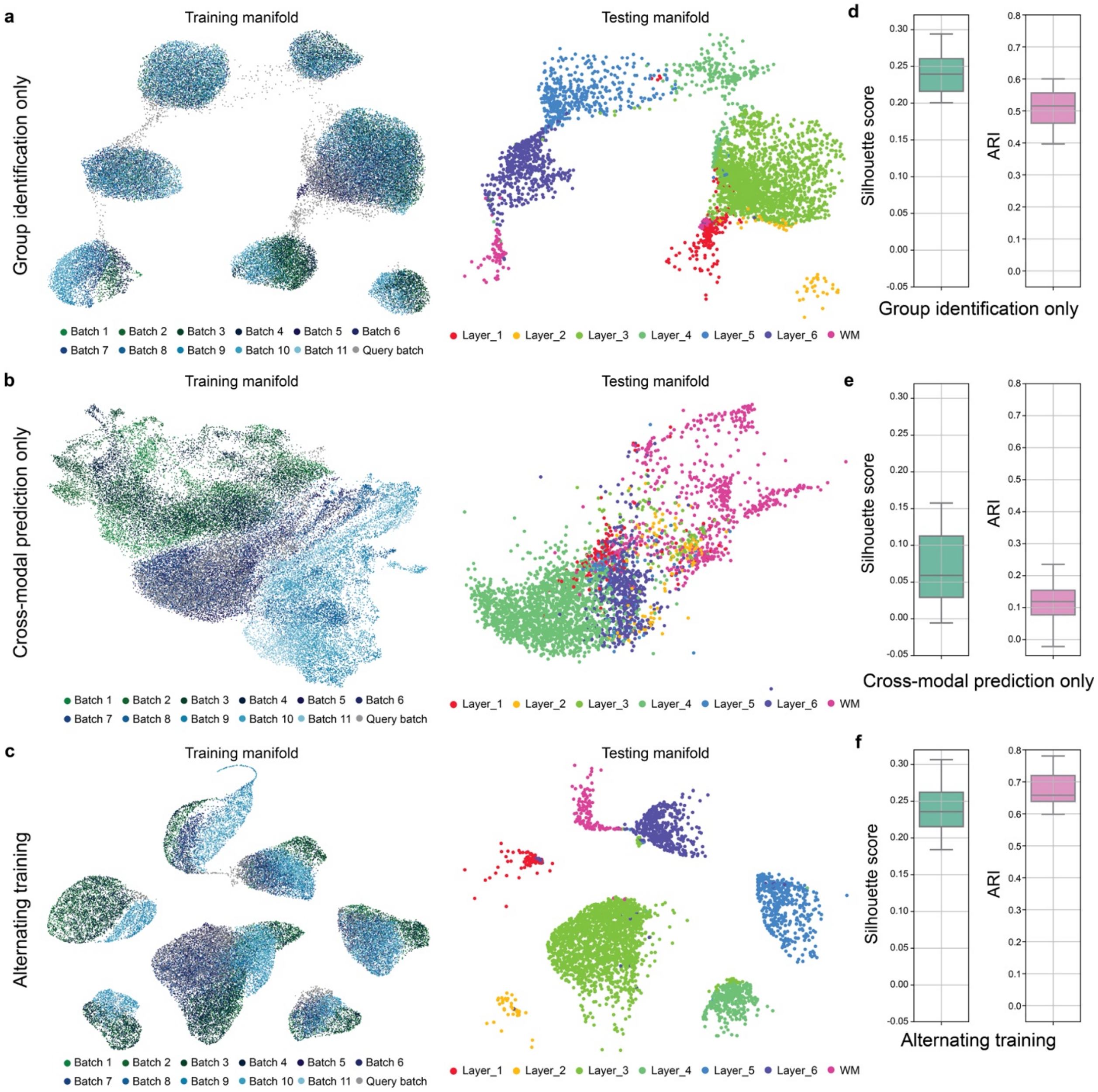

Supplementary Fig. 18: Batch effect correction in spatial DLPFC dataset with UnitedNet. a-c, The UMAP latent space visualizations showing shared latent codes of UnitedNet trained with group identification task only (**a**), cross-modal prediction task only (**b**), and alternating training (**c**) of the 12-batch spatial DLPFC dataset. In the training manifold, the latent codes are colored by different batches. In the testing manifold, the latent codes are colored by cell-type annotations. **d-f**, Boxplot showing the comparison of testing manifold separability in terms of Silhouette score and testing data group identification accuracy in terms of ARI between the UnitedNet trained by joint group identification task only (**d**), cross-modal prediction task only (**e**), and alternating training (**f**) for the spatial DLPFC dataset. n = 12 folds cross-validation for panel **d-f**. Box, 75%

and 25% quantiles. Line, median. Whisker, the maxima/minima or to the median $\pm 1.5\times$ IQR. Source data are provided as a Source Data file.
