# Supplementary material for: Explainable multi-task learning for multi-modality biological data analysis
Source: Nat Commun. 2023 May 3;14:2546. doi: 10.1038/s41467-023-37477-x (PMC10156823; doi:10.1038/s41467-023-37477-x)
Supplement: Supplementary file 3 — Reporting Summary [file 41467_2023_37477_MOESM3_ESM.pdf]

nature portfolio

Corresponding author(s): Jia Liu, Jie Ding

Last updated by author(s): Feb 27, 2023

Reporting Summary

Nature Portfolio wishes to improve the reproducibility of the work that we publish. This form provides structure for consistency and transparency in reporting. For further information on Nature Portfolio policies, see our Editorial Policies and the Editorial Policy Checklist.

Statistics

For all statistical analyses, confirm that the following items are present in the figure legend, table legend, main text, or Methods section.

n/a	Confirmed	
☐	☒	The exact sample size (*n*) for each experimental group/condition, given as a discrete number and unit of measurement
☐	☒	A statement on whether measurements were taken from distinct samples or whether the same sample was measured repeatedly
☐	☒	The statistical test(s) used AND whether they are one- or two-sided<br>*Only common tests should be described solely by name; describe more complex techniques in the Methods section.*
☒	☐	A description of all covariates tested
☐	☒	A description of any assumptions or corrections, such as tests of normality and adjustment for multiple comparisons
☐	☒	A full description of the statistical parameters including central tendency (e.g. means) or other basic estimates (e.g. regression coefficient) AND variation (e.g. standard deviation) or associated estimates of uncertainty (e.g. confidence intervals)
☐	☒	For null hypothesis testing, the test statistic (e.g. *F*, *t*, *r*) with confidence intervals, effect sizes, degrees of freedom and *P* value noted<br>*Give P values as exact values whenever suitable.*
☒	☐	For Bayesian analysis, information on the choice of priors and Markov chain Monte Carlo settings
☒	☐	For hierarchical and complex designs, identification of the appropriate level for tests and full reporting of outcomes
☐	☒	Estimates of effect sizes (e.g. Cohen's *d*, Pearson's *r*), indicating how they were calculated

Our web collection on statistics for biologists contains articles on many of the points above.

Software and code

Policy information about availability of computer code

Data collection	No software was used for data collection.
Data analysis	All data analysis and visualization in this study are implemented based on Python 3.7. The following packages and software were used: Jupyter 1.0.0, Anaconda 4.10.3, Leiden 0.8.9, UMAP 0.5.2 (https://github.com/lmcinnes/umap), AllenSDK 2.10.1 (https://github.com/AllenInstitute/AllenSDK), torch 1.11.0 (https://pytorch.org/), Scanpy 1.7.3, h5py 3.6.0, anndata 0.8.0, scikit-learn 0.24.2, matplotlib 3.5.1, seaborn 0.11.2, mne 1.0.3, mne-connectivity 0.3, numpy 1.21.5, scipy 1.7.3, pandas 1.3.5, pickle5 0.0.12. Custom code used in this study has been deposited on GitHub (https://github.com/LiuLab-Bioelectronics-Harvard/UnitedNet) and Zenodo (10.5281/zenodo.7679366).

For manuscripts utilizing custom algorithms or software that are central to the research but not yet described in published literature, software must be made available to editors and reviewers. We strongly encourage code deposition in a community repository (e.g. GitHub). See the Nature Portfolio guidelines for submitting code & software for further information.

Data

Policy information about availability of data

All manuscripts must include a data availability statement. This statement should provide the following information, where applicable:

- Accession codes, unique identifiers, or web links for publicly available datasets
- A description of any restrictions on data availability
- For clinical datasets or third party data, please ensure that the statement adheres to our policy

The Dygen simulation data used in this study are available in the https://github.com/dynverse/dyngen. The MUSE simulation data used in this study are available in

the https://github.com/AltschulerWu-Lab/MUSE. The Patch-seq GABAergic neuron dataset used in this study are available in the https://github.com/AllenInstitute/coupledAE-patchseq and https://knowledge.brain-map.org/data/1HEYEW7GMUKWIQW37BO/collections. The multiome ATAC+gene expression BMMC dataset used in this study are available in the https://openproblems.bio/neurips. The DBiT-dataset used in this study are available in the https://www.ncbi.nlm.nih.gov/geo/query/acc.cgi?acc=GSE137986. The DLPFC dataset used in this study are available in the https://doi.org/10.18112/openneuro.ds002076.v1.0.1. Source data are provided with this paper.

Human research participants

Policy information about studies involving human research participants and Sex and Gender in Research.

Reporting on sex and gender	N/A
Population characteristics	N/A
Recruitment	N/A
Ethics oversight	N/A

Note that full information on the approval of the study protocol must also be provided in the manuscript.

Field-specific reporting

Please select the one below that is the best fit for your research. If you are not sure, read the appropriate sections before making your selection.

[x] Life sciences [ ] Behavioural & social sciences [ ] Ecological, evolutionary & environmental sciences

For a reference copy of the document with all sections, see nature.com/documents/nr-reporting-summary-flat.pdf

Life sciences study design

All studies must disclose on these points even when the disclosure is negative.

Sample size	Sample sizes were estimated based on previous studies.<br>[He, Y. et al. ClusterMap for multi-scale clustering analysis of spatial gene expression. Nat. Commun. 12, 1-13 (2021).]<br>[Butler, A., Hoffman, P., Smibert, P., Papalexi, E. & Satija, R. Integrating single-cell transcriptomic data across different conditions, technologies, and species. Nat. Biotechnol. 36, 411-420, doi:10.1038/nbt.4096 (2018).]<br>[Gala, R. et al. Consistent cross-modal identification of cortical neurons with coupled autoencoders. Nat. Comput. Sci. 1, 120-127, doi:10.1038/s43588-021-00030-1 (2021).]<br>[Gayoso, A. et al. Joint probabilistic modeling of single-cell multi-omic data with totalVI. Nat. Methods 18, 272-282 (2021).]
Data exclusions	N/A. No data was excluded from the study.
Replication	All of the findings reported in this study are reproducible based on the code in the UnitedNet Github repository. All experimental procedures are detailed in the Methods to ensure reproducibility.
Randomization	Each fold of the cross validation was randomly spitted from the dataset or used as the existing biological group identities.
Blinding	N/A. Blinding was not relevant to the multi-modal data analyses because the datasets were not divided into control and experimental groups.

Reporting for specific materials, systems and methods

We require information from authors about some types of materials, experimental systems and methods used in many studies. Here, indicate whether each material, system or method listed is relevant to your study. If you are not sure if a list item applies to your research, read the appropriate section before selecting a response.

Materials & experimental systems

n/a	Involved in the study
[x]	[ ] Antibodies
[x]	[ ] Eukaryotic cell lines
[x]	[ ] Palaeontology and archaeology
[x]	[ ] Animals and other organisms
[x]	[ ] Clinical data
[x]	[ ] Dual use research of concern

Methods

n/a	Involved in the study
[x]	[ ] ChIP-seq
[x]	[ ] Flow cytometry
[x]	[ ] MRI-based neuroimaging
